# Traction Radiculopathy After Surgery for Lumbar Spinal Metastasis: A Case Report

**DOI:** 10.7759/cureus.28809

**Published:** 2022-09-05

**Authors:** Toshiki Ishibashi, Ryoma Aoyama, Hiraku Hotta, Itsuo Watanabe, Yuichiro Takahashi, Shogo Matsumoto, Ryosuke Yamaki, Ukei Anazawa

**Affiliations:** 1 Orthopaedics, Tokyo Dental College Ichikawa General Hospital, Ichikawa, JPN

**Keywords:** herniated tumor, locomotive syndrome, postoperative palsy, traction radiculopathy, metastatic spinal tumor

## Abstract

Treatment of spinal metastasis has attracted much attention globally, especially in Japan, with the advancement of cancer therapy. Among the metastases, those from breast and prostate cancers may be more important than others considering the high incidence of bone metastasis and the long-term prognosis. This condition often results in surgical procedures of spinal metastases to improve cancer patients' quality of life (QOL).

In the present case, a patient with lumbar metastasis of breast cancer presented with right L5 nerve palsy after palliative laminectomy surgery with posterior fusion. The nerve palsy had improved after additional bone resection around the right L5 root.

The mechanism of this postoperative leg paralysis was subclinical nerve root damage due to the narrowing of the intervertebral foramen caused by the tumor protrusion like lumber disc hernia and the stretching of the nerve roots caused by the posterior shift of the dural tube.

When performing decompression and fixation of a metastatic spine showing a herniated tumor formed by a tumor protruding posteriorly into the intervertebral foraminal space, sufficient tumor mass debulking should be considered to avoid postoperative intervertebral foraminal stenosis.

## Introduction

Recently, the opportunity for the treatment of metastatic spinal tumors has been increasing as the prognosis of cancer patients improved due to advances in cancer treatment [1-3]. Breast cancer and prostate cancer that are characteristic of a high frequency of bone metastasis have a good prognosis, and surgical treatment for spinal metastases is increasingly considered during treatment [2,3].

In this study, we experienced a patient with breast cancer who presented with lumbar pain and L5 radicular pain due to solitary lumbar metastasis. The patient underwent laminectomy with posterior lumbar fusion surgery and presented with L5 nerve palsy on the first day after surgery. The patient underwent emergency surgery again to decompress the nerve root, and the paralysis gradually improved.

In this case, the mechanism of postoperative leg paralysis was subclinical nerve root damage due to the narrowing of the intervertebral foramen caused by the herniated tumor, which is a posterior protrusion of the tumor, and the stretching of the nerve root caused by the posterior shift of the dural tube. We believe that the mechanism of paralysis, in this case, could be predictable before surgery by radiological images and that the discussion of this case will provide useful information for future surgeries for metastatic spinal tumors.

## Case presentation

**Figure 1:**
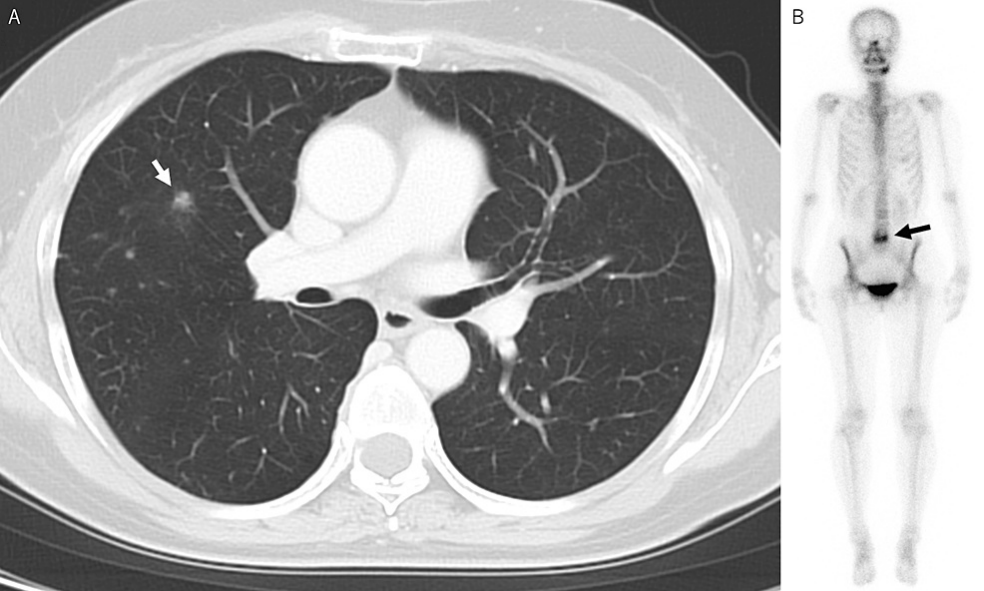
(A) Axial CT image of the chest at the initial examination. (B) Whole body bone scintigraphy at the initial examination. A. Metastatic lung tumor was seen in the right thoracic region (arrow). B. There was an accumulation in the L5 vertebral body, and a multisite metastatic bone tumor was not evident.

**Figure 2:**
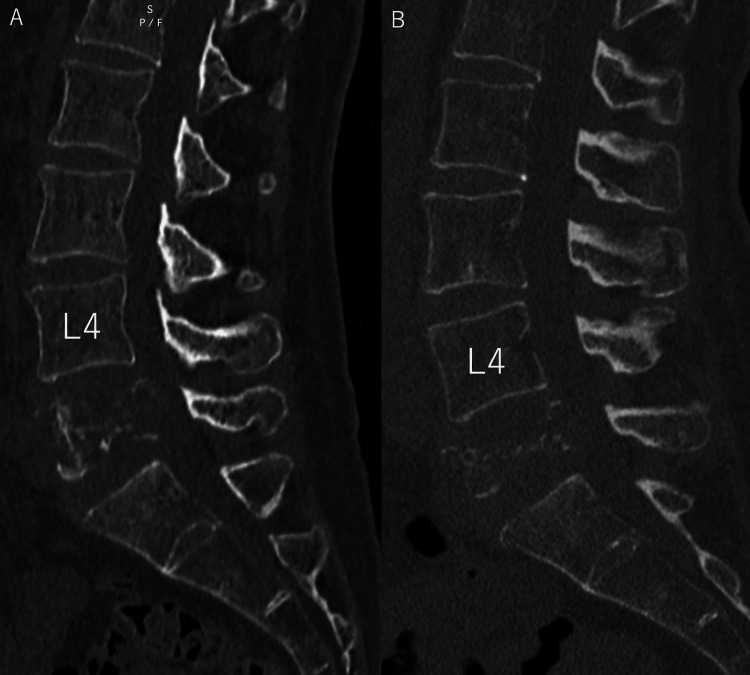
(A) Sagittal CT image at initial examination. (B) Sagittal CT image one month after the initial examination. A. The L5 vertebral body showed osteolytic changes and was considered a metastatic spinal tumor. B. The L5 vertebral body showed progressive destruction and osteolytic changes.

**Figure 3:**
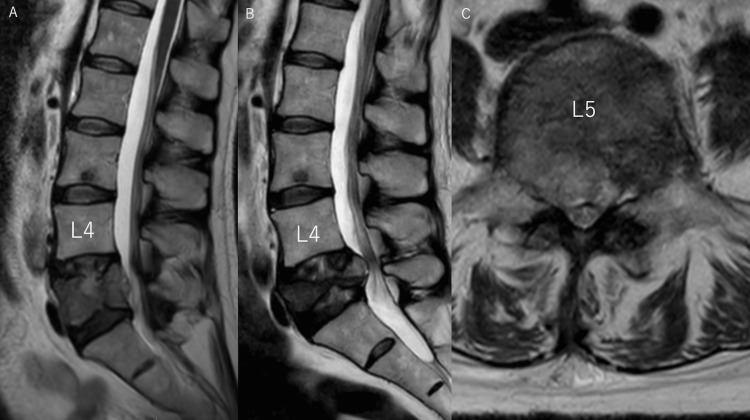
(A) T2-weighted MRI sagittal section at the initial examination. (B) T2-weighted MRI sagittal section one month after the initial examination. (C) T2-weighted MRI transverse section one month after the initial examination. A. High-intensity tumor was seen at the center of the L5 vertebral body and in the epidural region. B. The L5 vertebral body showed progressive collapse, and the high-intensity tumor protruded posteriorly, presenting spinal canal stenosis. C. The L5 vertebral body showed a significant high-intensity tumor extending the epidural space predominantly on the right side. This protrusion resulted in severe dural canal compression and intervertebral foraminal stenosis.

A 51-year-old woman diagnosed with Stage 4 breast cancer with multiple lung metastases visited our hospital in July 2011. Hormonal therapy with anastrozole and leuprorelin acetate resulted in shrinkage of the primary lesion and pulmonary metastases. The disease progression had been controlled with hormone therapy. In December 2020, she fell on her buttocks and developed back pain; in January 2021, this symptom made her hard to walk. MRI, CT, and bone scintigraphy revealed metastasis of the L5 vertebra (Figures 1-3). She was consulted to our department for treating this metastasis in February 2021. There was no history of disease except breast cancer.

At the initial examination, she was found to have marked low back pain with body movement, accompanied by lower extremity pain in the right L5 region upon awakening. There were no abnormal neurological findings. Treatment of bone modifying drug, denosumab 120 mg a month, was initiated for control of L5 metastasis. Despite denosumab administration, MRI and CT revealed progressive tumor disruption (Figures 2, 3). The pain with movement remained intolerable two months after her first visit to our department. Therefore, she underwent decompression between L4/5 and L5/S with a posterior fusion between L4 and S1. Intraoperatively, the tumor protruding into the epidural space inside the spinal canal was partially removed, and unroofing of the L5 and S1 nerve routes was performed simultaneously. There was no complication of intraoperative neurological injury. Pathologically, this tumor was confirmed to be breast cancer.

**Figure 4:**
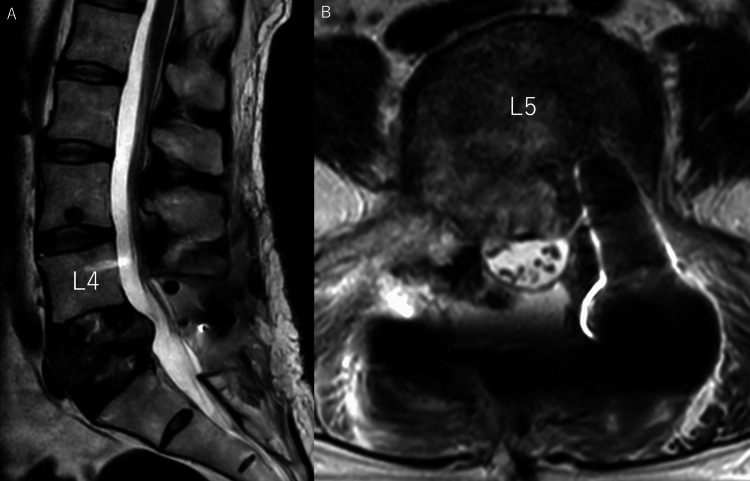
(A) T2-weighted MRI sagittal section, the first day after the surgery. (B) T2-weighted MRI transverse section, the first day after the surgery. A. The L5 vertebral canal stenosis at the L5 vertebral body level was removed by L5 laminectomy. A hematoma was seen posterior to the dural canal, but it did not compress the dural tube. B. A pedicle screw was found to have no signal area at the left L5 pedicle. The dural canal was well decompressed, though the dorsal tumor protrusion remained, while the L5 nerve root was not decompressed at the lateral side.

On the first day after surgery, muscle weakness appeared mainly in the right L5 area, and the gluteus medius, tibialis anterior, and extensor hallucis longus muscles manifested weakness (Manual muscle testing (MMT): 3). MMT is graded from 0 to 5: 0, zero; 1, trace; 2, poor; 3, fair; 4, good; 5, normal. An urgent MRI showed a satisfactory decompression of the dural tube with remained hematoma and metastatic tumor (Figure 4). To clarify the cause of paralysis, we performed emergency surgery to confirm the L5 nerve root condition. Intraoperative findings revealed no nerve compression due to hematoma or protruding screws. There was no posterior deviation of the tumor inside the spinal canal. The nerve root was erythematous and tense with a posterior shift of the dura tube. The cause of nerve root palsy seemed to be intervertebral foraminal stenosis due to a residual herniated tumor on the lateral side outside the spinal canal. Additional bone resection around the right L5 root was performed.

**Figure 5:**
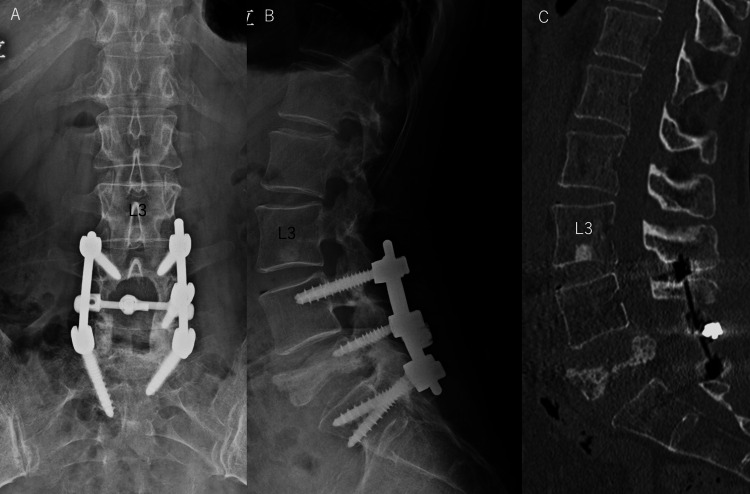
(A) A plain lumbar spine anteroposterior X-ray image at one year postoperatively. (B) A lateral plain lumbar spine X-ray image at one year postoperatively. (C) Sagittal CT image at 11 months postoperatively. A. There was no implant loosening, and good alignment was well maintained. B. Sclerotic change of L5 vertebral body was revealed, and good alignment was well maintained. C. Sclerotic change of L5 vertebral body was confirmed, suggesting well tumor control, and the spinal canal was enlarged.

Postoperatively, a total of 24 Gy of palliative irradiation was performed on the metastatic area of the L5 vertebra. Muscle weakness in the right L5 area gradually improved after the second surgery, and muscle strength was almost restored to MMT: 5 in four months. She was able to return to work as a childcare worker without any activity of daily living (ADL) impairment. Postoperatively, denosumab continued to be administered. One year after surgery, she was unaware of lower back and leg pain, and only mild numbness in her lower limbs remained. She continued to work without any problems. One year after surgery, plain X-rays and CT scans demonstrated the bone sclerotic change without tumor progression, indicating good tumor control due to hormone therapy and bone modifying drug with radiotherapy (Figure 5).

## Discussion

In Japan, where 29.0% of the population is over 65 years old, cancer is the leading cause of death, accounting for 26.5% of all deaths [4,5]. Recent advances in cancer treatment and other fields of medicine have improved the prognosis for cancer patients, and more and more patients are living with cancer for a long period [2,3]. The Japanese Orthopedic Association has even proposed the concept of "locomotive syndrome in cancer patients," and treating locomotor diseases in cancer patients is an urgent issue that orthopedic surgeons in Japan need to address [6]. For this reason, opportunities for treating metastatic spinal tumors are also increasing [2]. In particular, breast and prostate cancer have a long prognosis, and surgical treatment of spinal metastases is increasingly considered during treatment [2,3].

In this case, the patient was diagnosed with stage 4 breast cancer, but hormonal therapy was effective enough to slow the progression of the primary tumor, and no new metastatic lesion had appeared for 10 years. After a long course of cancer, walking disability appeared due to L5 metastasis. This case can be considered a typical example of locomotive syndrome in cancer patients in modern Japan, in which the patient required locomotor treatment due to the long-term course of cancer. In our case, the lumbar metastases are well controlled without changing breast cancer status postoperatively, and ADL disability has completely disappeared. As described above, in this era of advanced cancer treatment, the treatment of metastatic tumors also plays an important role in improving the ADL of cancer patients.

This case demonstrates the importance of predicting postoperative traction radiculopathy in surgery for metastatic spinal tumors with herniated tumor protruding into lateral foraminal space.

Traction radiculopathy is a nerve root disorder that appears after posterior spinal decompression, as Tsuzuki reported [7]. He performed an anatomical study using cadavers and found that nerve root damage was caused by the posterior traction of nerve roots due to the posterior expansion of the dura mater after posterior decompression.

To the best of our knowledge, this is the first report of postoperative neuropathy due to traction radiculopathy for metastatic spinal tumors. On the other hand, reports of nerve palsy after lumbar spine surgery for the degenerative disease are relatively common, the incidence of which has been reported to range from 0.5% to 24% [8,9]. However, the causes of nerve palsy were rarely investigated. A detailed study of the causes of neuropathy after posterior lumbar fusion demonstrated that 56% of neuropathy cases were associated with postoperative nerve root erythema and relative nerve root stenosis, which are similar to our spinal metastasis case. This report also postulated that the narrowing of the intervertebral foramen is the cause of postoperative paralysis [9]. However, there are few reports of traction radiculopathy in the lumbar spine, and the mechanism is poorly understood.

**Figure 6:**
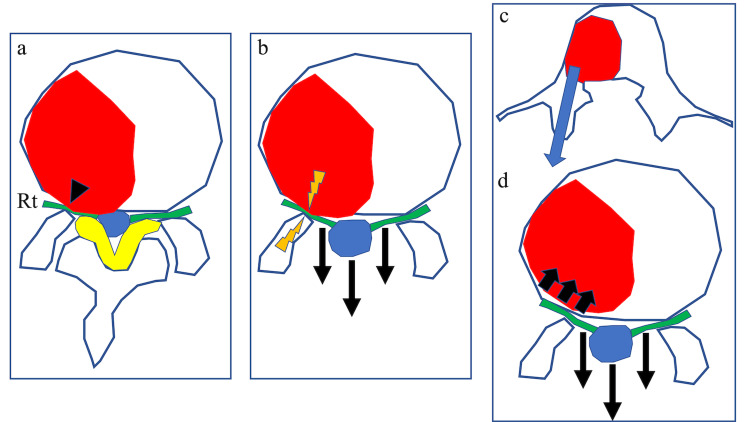
A schematic illustration of traction radiculopathy due to intervertebral foraminal stenosis caused by a herniated tumor a. In the right intervertebral foramen, there is compression of the nerve root by the tumor (arrowhead). The dural tube is compressed by the yellow ligament and tumor. b. Laminectomy and flavectomy enlarge the dural tube and causes a posterior shift of the dural tube. The nerve root is tractioned posteriorly, and traction and compression of the nerve root occur simultaneously. Intervertebral foraminal stenosis due to herniated tumor induces traction radiculopathy after decompression. c. Transpedicular tumor debulking can reduce the compression of the right nerve root in the intervertebral foramen. d. If the intervertebral foraminal stenosis is relieved by debulking tumor, traction radiculopathy is unlikely to happen even if the nerve root is tractioned after decompression.

In contrast, many reports of C5 palsy have been reported after cervical surgery [10-12]. Many have suggested that intervertebral foraminal stenosis or traction radiculopathy may be involved in postoperative C5 palsy of the cervical spine. However, the exact mechanism is still unclear, and the disease is considered multifactorial [10]. Therefore, intervertebral foraminal stenosis and nerve stretching in the lumbar spine might be involved in postoperative nerve root paralysis similar to a possible mechanism of C5 palsy (Figure 6).

Taken together, when surgeons perform palliative surgery for lumbar spinal metastasis, they need to consider the following points: 1) degree of compression around the nerve root at the intervertebral foramen should be evaluated radiographically, 2) if there is a nerve root compression at the intervertebral foramen, surgeons should consider a surgical decompression around the nerve root to avoid postoperative paralysis even the patients did not have preoperative palsy. On the other hand, the vertebrae body with the tumor are fragile, and easy bony decompression of the intervertebral foramen may cause strong spinal instability. Therefore, surgeons need to consider the addition of spinal instrumental fixation for such cases to achieve satisfactory stability.

Therefore, if the nerve root compression at the intervertebral foramen is anticipated due to the herniated tumor extending laterally into the epidural space, it is desirable to shrink the tumor without destroying the bony elements of the intervertebral foramen.

Especially in lumber metastasis cases with severe nerve root pain due to the herniated tumor, it is essential to debulk the tumor adequately around the nerve root. In addition, we previously reported minimally invasive tumor reduction techniques such as transpedicular tumor resection [13]. Since the tumor is removed through a transpedicular approach in this technique, the bony hole for inserting a curette longueur is small, and bleeding can be easily controlled by placing bone wax at the bony hole. This technique also has the advantage of reducing postoperative bleeding from the tumor. Transpedicular tumor resection may be useful as a minimally invasive procedure to relieve nerve compression at the intervertebral foramen (Figure 6).

## Conclusions

In Japan, where advances in cancer treatment have been made, the control of metastatic tumors in cancer patients is becoming increasingly important. In particular, the demand for control of metastatic spinal tumors is increasing. In the present study, we reported a breast cancer patient with traction radiculopathy caused by residual herniated tumor after posterior lumbar decompression and fusion surgery.

When performing decompression of metastatic spinal tumors with intervertebral foraminal stenosis caused by herniated tumor, minimally invasive tumor reduction techniques such as transpedicular tumor resection should be considered to avoid traction radiculopathy.
